# Epidemiological Profile of High-Risk HPV in Cervical Samples for Liquid-Based Cytology: Insights from Screening and Symptomatic Cohorts

**DOI:** 10.3390/microorganisms14081794

**Published:** 2026-08-14

**Authors:** Antoanela Curici, Denitsa Tsaneva-Damyanova, Gergana Nedelcheva, Luciana Alexandra Pavelescu

**Affiliations:** 1 Department of Cellular and Molecular Biology and Histology, “Carol Davila” University of Medicine and Pharmacy, 050474 Bucharest, Romania; antoanela.curici@umfcd.ro (A.C.); luciana.pavelescu@umfcd.ro (L.A.P.); 2 Synevo Romania, 021408 Bucharest, Romania; 3 Department of Microbiology and Virology, Medical University, 3 Bregalnitsa St., 9000 Varna, Bulgaria; gergana.kuyumdzhieva@mu-varna.bg; 4 Medical Diagnostics Laboratory Synevo Bulgaria, 24 Saborni Blvd., 9000 Varna, Bulgaria

**Keywords:** human papillomavirus, high-risk HPV genotypes, liquid-based cytology, Bulgaria, cervical cancer

## Abstract

High-risk human papillomavirus (HR-HPV) infection is the principal etiological factor in cervical cancer, with genotype distribution and cytological associations varying across populations. This retrospective study aimed to characterize the prevalence and genotype distribution of HR-HPV among 1747 Bulgarian women, including a subgroup of 1134 women with paired HPV polymerase chain reaction (PCR) and liquid-based cytology (LBC) results. The study population comprised women undergoing routine cervical screening (76.0%, *n* = 1328) and women tested because of clinical indications, including gynecological symptoms (24.0%, *n* = 419). The overall HR-HPV prevalence was 31.4% (549/1747) and was significantly higher among women tested for clinical indications than among those undergoing routine screening. HPV16 was the most frequently detected genotype, followed by multiple HR-HPV infections and other genotypes, including HPV31, HPV51, HPV66, HPV18, HPV45, HPV56, HPV68, and HPV33. Cytological abnormalities were identified in 24.7% (280/1134) of women with paired HPV and cytology results. HR-HPV positivity was strongly associated with abnormal cytology; however, 27.3% (233/854) of women with negative cytology were also HR-HPV positive. Integrating HPV genotyping with cytology may improve risk stratification and support more targeted clinical management. Despite the increasing use of LBC and HPV co-testing, comparative data describing HR-HPV genotype distribution and its relationship with cytological findings in screening and symptomatic populations remain limited. These findings provide epidemiological evidence on HR-HPV infection patterns in Bulgarian women and establish a foundation for future studies evaluating genotype-specific risks, disease progression, and population-based surveillance strategies.

## 1. Introduction

Human papillomavirus (HPV) contains a circular, double-stranded DNA genome that encodes several viral proteins crucial for its replication and pathogenicity [1]. Approximately 40 types of HPV specifically target the mucosal epithelium. Among these, HPV16, HPV18, HPV31, HPV33, HPV35, HPV39, HPV45, HPV51, HPV52, HPV53, HPV56, HPV58, HPV59, HPV66, and HPV68 are classified as high-risk oncogenic types [2]. While most HPV infections are transient and are resolved by the host immune system within 1–2 years, persistent infection with oncogenic genotypes can lead to progressive epithelial abnormalities and malignant transformation, posing a significant global public health issue [3]. Cervical cancer remains one of the most prevalent cancers among women, especially in low- and middle-income countries, where organized screening programs and HPV vaccination coverage are often inadequate. The carcinogenic potential of high-risk HPV is attributed to the viral E6 and E7 oncoproteins, which promote uncontrolled cell proliferation and inhibit apoptosis by inactivating cell cycle control factors. Specifically, E6 facilitates the degradation of the p53 tumor suppressor protein, thereby preventing apoptosis and allowing genomic instability, while E7 inactivates the retinoblastoma protein (pRb), leading to unregulated cell cycle progression. Collectively, these viral oncogenes enable cells to bypass normal growth controls and facilitate malignant transformation, making them significant targets for therapeutic interventions. Additionally, the functional variations and polymorphisms in the *E6* and *E7* genes across different HPV types and variants influence their oncogenic potential and clinical outcomes [4].

Information regarding the epidemiology of high-risk HPV in South-Eastern Europe is still limited. Studies indicate that the prevalence and genotype distribution of high-risk human papillomavirus in women are affected by variables including age, sexual practices, immune health, geographic region, and the clinical circumstances under which testing occurs. Although HPV16 remains the most prevalent genotype globally, the occurrence of other oncogenic genotypes, including HPV31, HPV33, HPV45, HPV52, HPV58, and HPV66, varies significantly among different populations and regions [5]. In a substantial Romanian cohort comprising 82,513 women, Curici and colleagues identified HPV16 as the most prevalent genotype linked to cytological abnormalities. They also highlighted significant regional variations in the prevalence of non-HPV16/HPV18 high-risk HPV genotypes [6]. Currently, there is a significant gap in comprehensive data concerning the effects of HR-HPV on the Bulgarian population, which is vital for crafting effective prevention strategies to curb HPV transmission within the country. Consequently, country-specific epidemiological data are crucial for the optimization of screening algorithms, risk stratification, and vaccination strategies. Typically, screening groups, which consist of asymptomatic women undergoing routine examinations, may display distinct HR-HPV profiles compared to symptomatic women, who might already exhibit cytological abnormalities or underlying lesions [7].

Molecular testing for high-risk human papillomavirus is advocated as either an adjunctive or primary approach for cervical cancer screening, owing to its enhanced sensitivity relative to cytology alone [8]. Liquid-based cytology (LBC) has increasingly replaced conventional Pap smears in numerous screening programs, attributed to its superior specimen adequacy, standardized processing, and compatibility with ancillary molecular testing, including HPV DNA detection and genotyping. The integration of HR-HPV testing into LBC-based cervical screening has augmented the sensitivity for identifying high-grade lesions and has enabled risk stratification based on viral genotype and viral load [9]. Additionally, the association between specific HR-HPV genotypes and cytological findings in liquid-based cytology can elucidate the relative oncogenic potential of various types, thereby informing the development of triage algorithms, follow-up intervals, and vaccination strategies. Simultaneously, LBC samples obtained from women exhibiting gynecological symptoms (such as abnormal bleeding or discharge) offer an opportunity to compare HPV infection patterns between asymptomatic screening populations and clinically symptomatic cohorts [10].

While the adoption of LBC and HPV PCR co-testing is on the rise, there remains a notable deficiency in comparative data regarding HR-HPV infection patterns between screening and symptomatic populations across various regions. It is imperative to conduct studies that simultaneously examine HR-HPV prevalence, genotype distribution, and associated cytological abnormalities in these two distinct yet complementary clinical groups [11]. Such evidence is crucial for developing more targeted screening recommendations, optimizing healthcare resource allocation, and improving the identification of women at the highest risk for high-grade lesions and cervical cancer.

This research sought to investigate the prevalence and genotype distribution of 14 high-risk human papillomavirus infections among Bulgarian women, specifically HPV16, HPV18, HPV31, HPV33, HPV35, HPV39, HPV45, HPV51, HPV52, HPV56, HPV58, HPV59, HPV66, and HPV68. It also aimed to assess the link between HR-HPV infections and abnormalities identified via liquid-based cytology. The study involved women who were either undergoing routine screenings or presenting with gynecological symptoms and had liquid-based cytology tests. The research concentrated on age-related patterns, the presence of multiple HPV infections, and the relationship between HPV genotypes and cervical lesions hypotheses. The results are intended to provide evidence that could improve risk-based screening strategies and enhance cervical cancer prevention efforts.

## 2. Materials and Methods

### 2.1. Study Design and Population

This retrospective, cross-sectional study included women who underwent high-risk human papillomavirus (HR-HPV) testing within the Synevo laboratory network between January 2024 and June 2026. The study included all eligible women tested during this period for whom complete laboratory HPV PCR records were available. No additional sampling procedure was applied.

A total of 1747 women were included in the overall HR-HPV prevalence and genotype-distribution analysis. HR-HPV testing was performed using either the Seegene Anyplex^TM^ II HPV28 Detection assay (Seegene Inc., Seoul, Republic of Korea; *n* = 1272) or the Abbott Alinity m HR-HPV assay (Abbott Molecular Inc., Des Plaines, IL, USA; *n* = 475).

Of the 1747 women included in the HPV analysis, 613 women did not have a corresponding Pap cytology result available and were excluded from the combined cytology–HPV analysis. The remaining 1134 women had paired HR-HPV PCR and liquid-based cytology results and were included in the cytology-associated analyses. These paired samples were distributed as follows:

• N = 871 women with cytology testing and HPV PCR on Seegene platform; SurePath technique liquid-based cytology.
• N = 263 women with cytology testing and HPV PCR on Alinity m platform; ThinPrep technique liquid-based cytology.

The study population comprised two clinically relevant groups: women undergoing routine cervical screening and women tested because of clinical indications. The routine screening group included women attending scheduled cervical cancer screening or preventive gynecological check-ups without documented symptoms at the time of testing. The clinical-indication group included women referred for HPV testing because of gynecological symptoms, such as abnormal vaginal bleeding, pelvic pain, or visible cervical lesions, as well as women undergoing follow-up evaluation after previously detected HPV infection, abnormal cytology, abnormal colposcopic findings, or other clinician-directed diagnostic indications. Thus, the clinical-indication group included both symptomatic women and asymptomatic women undergoing follow-up for prior abnormalities.

Women undergoing routine screening and those tested for clinical indications may differ in baseline characteristics, screening history, referral pathways, previous HPV status, cytology history, vaccination status, and treatment history. Because these variables were not consistently available in the retrospective laboratory dataset, adjustment for potential confounding or selection bias was limited. This limitation was considered when interpreting differences between the two groups and is further addressed in the Discussion section. We suggest collecting and integrating these clinical and epidemiological variables in future studies to enable proper stratification or multivariable adjustment, minimizing confounding and improving estimate precision.

### 2.2. Ethical Considerations

The research adhered to the guidelines set by the Declaration of Helsinki and was approved by the Synevo Ethics Committee (approval code: SYN 2/April 2026). Participants gave written informed consent for testing at the Synevo Clinical Laboratory, where medical ethics principles and European legal standards were explicitly communicated. For participants under 18, parental consent was obtained according to institutional protocols, and adolescents received age-appropriate information to ensure their assent and confirm their voluntary participation. The informed consent process thoroughly informed participants about the nature, purpose, risks, benefits, and alternatives of HPV PCR and cytology testing. No directly identifiable personal information was included in the analysis or reported in the manuscript.

### 2.3. Sample Collection and Processing

Cervical samples were collected by gynecologists during routine screening visits, diagnostic consultations, or follow-up examinations. Samples were obtained using a cervical sampling brush and transferred into liquid-based cytology collection media suitable for both cytological evaluation and molecular HPV testing.

Two laboratory workflows were used according to the collection medium and molecular platform.

#### 2.3.1. Subgroup 1: Seegene Platform and SurePath Liquid-Based Cytology (*n* = 871)

In subgroup 1, cervical samples were collected using a specialized cervical brush and transferred into SurePath liquid-based cytology containers. Samples were stored and transported to the laboratory under conditions recommended for the SurePath technique.

Liquid-based cytology was performed using the BD SurePath^TM^ method. HR-HPV genotyping was performed using the Seegene Anyplex^TM^ II HPV28 Detection assay. This subgroup included 871 women with paired HPV PCR and cytology results.

The SurePath preservation medium maintains cellular integrity and allows cytological evaluation from the collected cervical specimen. Processing and interpretation were performed according to standard laboratory procedures and manufacturer recommendations.

#### 2.3.2. Subgroup 2: Alinity m Platform and ThinPrep Liquid-Based Cytology (*n* = 263)

In subgroup 2, cervical samples were collected using a specialized cervical brush and placed into ThinPrep PreservCyt^®^ collection vials (Hologic, Inc., Marlborough, MA, USA). Samples were stored at room temperature and transported to the laboratory for processing.

Upon arrival at Synevo, samples collected in PreservCyt solution were used for concurrent HR-HPV PCR testing and liquid-based cytology, when paired testing was requested. This subgroup included 263 women with paired HPV PCR and cytology results.

The PreservCyt solution is designed to preserve cellular material for cytological assessment and ancillary molecular testing. Sample processing, HPV testing, and cytological evaluation were performed according to manufacturer instructions and routine laboratory protocols.

### 2.4. Cervical Cytology

Liquid-based cytology slides were prepared from the same cervical collection vial used for HPV testing whenever paired testing was available. SurePath liquid-based cytology was used for subgroup 1, and ThinPrep liquid-based cytology was used for subgroup 2.

Cytology slides were evaluated by cytopathologists according to the 2014 Bethesda System for Reporting Cervical Cytology. Cytological evaluation was performed independently by cytopathologists who were blinded to HPV PCR results. System for reporting cervical cytology, classified as: negative for intraepithelial lesion or malignancy (NILM), atypical squamous cells of undetermined significance (ASC-US), atypical squamous cells cannot exclude HSIL (ASC-H), low-grade squamous intraepithelial lesion (LSIL), high-grade squamous intraepithelial lesion (HSIL), atypical glandular cells (AGC), and squamous cell carcinoma/adenocarcinoma. For statistical analyses, cytological findings were also grouped as negative cytology or abnormal cytology. Abnormal cytology included ASC-US, ASC-H, LSIL, HSIL, AGC, squamous cell carcinoma, and adenocarcinoma.

### 2.5. HR-HPV PCR Testing

#### 2.5.1. Subgroup 1—Seegene Anyplex^TM^ II HPV28 Assay

HPV genotyping in the subgroup 1 was performed using the Seegene Anyplex^TM^ II HPV28 Detection assay (Seegene Inc., Seoul, Republic of Korea), a semiquantitative multiplex real-time PCR assay that simultaneously detects and genotypes 28 HPV types across two reaction wells (primer sets A and B). The assay uses Seegene’s proprietary dual priming oligonucleotide (DPO^TM^) and tagging oligonucleotide cleavage and extension (TOCE^TM^) technologies, with genotype discrimination achieved through catcher melting temperature analysis (CMTA). The assay is designed to detect a total of 28 HPV genotypes, which are divided into two categories: 19 high-risk/probable high-risk HPV genotypes (HPV16, HPV18, HPV31, HPV33, HPV35, HPV39, HPV45, HPV51, HPV52, HPV53, HPV56, HPV58, HPV59, HPV66, HPV68, HPV69, HPV70, HPV73, HPV82) and 9 low-risk genotypes. The human β-globin gene was co-amplified as an endogenous internal control to confirm specimen adequacy. Reactions were performed and interpreted according to the manufacturer’s instructions for use.

#### 2.5.2. Subgroup 2—Abbott Alinity m HR HPV Assay

HPV genotyping in the subgroup 2 was performed using the Alinity m HR-HPV assay (Abbott Molecular Inc., Des Plaines, IL, USA) on the fully automated Alinity m System. This qualitative real-time PCR assay amplifies and detects DNA sequences from 14 HR-HPV genotypes. The assay provides detailed information on three primary high-risk HPV genotypes-HPV16, HPV18, and HPV45, and categorizes the remaining 11 targeted high-risk HPV genotypes into two groups: HR-HPV group A (HPV31, HPV33, HPV52, HPV58 genotypes) or HR-HPV group B (HPV35, HPV39, HPV51, HPV56, HPV59, HPV66, HPV68). Nucleic acid extraction was performed on-system using magnetic microparticle technology (Alinity m Sample Prep Kit), followed by real-time PCR amplification and fluorescence detection of both HR-HPV targets and the endogenous human β-globin internal control, according to the manufacturer’s instructions.

#### 2.5.3. Cross-Platform Comparability

Cross-platform comparability entails adopting a standardized framework that accounts for different genotyping panels and reporting styles, ensuring that data derived from distinct HPV assays can be pooled or compared meaningfully. This is usually achieved by harmonizing genotype results into common categories. Because the two subgroups were tested using different assays (Seegene Anyplex^TM^ II HPV28 vs. Abbott Alinity m HR-HPV) with partially overlapping but not identical genotype panels and reporting formats (individual HPV genotyping vs. aggregate group reporting for non-HPV16/HPV18/HPV45 types), HPV results were harmonized for the pooled analysis. We had classified results distribution of 14 distinct high-risk human papillomavirus genotypes: HPV16, HPV18, HPV31, HPV33, HPV35, HPV39, HPV45, HPV51, HPV52, HPV56, HPV58, HPV59, HPV66, and HPV68. Additionaly, we added two groups more for those samples processed on Alinity m: HR-HPV group A and HR-HPV group B. Therefore, genotype-level interpretation for individual types included within these aggregate Alinity m categories was limited. This limitation was considered when interpreting genotype-specific comparisons and is addressed in the Section 4.

Multiple HR-HPV infection was defined as detection of two or more HR-HPV genotypes or genotype groups in the same sample. In genotype-specific analyses, each detected genotype was counted independently. Therefore, genotype-specific categories were not mutually exclusive, and percentages across genotype categories were not expected to sum to 100%.

#### 2.5.4. Statistical Analysis

Statistical analyses were performed using the BrightStat.com software (https://secure.brightstat.com/index.php). Descriptive statistics, such as proportions and corresponding 95% confidence intervals (CI), were calculated. The prevalence of 14 high-risk HPV genotypes (HPV16, HPV18, HPV45, HPV31, HPV51, HPV52, HPV33, HPV58, HPV56, HPV59, HPV66, HPV35, HPV39, and HPV68) was assessed. In the testing of women using HR-HPV with Alinity m, aside from HPV16, HPV18, and HPV45, two further categories were identified: other HR-HPV A, which includes genotypes HPV31, 33, 52, and 58, and HR-HPV B, comprising HPV35, 39, 51, 56, 59, 66, and 68. For multiple HR-HPV infections, each detected HPV genotype was recorded independently. Women with two or more detected HR-HPV genotypes were classified as having multiple infections. In genotype-specific analyses, a woman with multiple infections contributed to the prevalence estimate of each genotype detected; therefore, genotype-specific categories were not mutually exclusive and percentages were not expected to sum to 100%. For genotype-specific association analyses, each genotype was coded as a binary variable (detected vs. not detected), irrespective of whether it occurred alone or in combination with other HR-HPV genotypes. Age data are presented as mean ± standard deviation and medians. Thirteen age groups (14–20, 21–25, 26–30, 31–35, 36–40, 41–45, 46–50, 51–55, 56–60, 61–65, 66–70, 71–75, >75) were categorized. The liquid-based cytology results for the majority of women were scored (*n* = 1134). The study examined the correlation between age, high-risk human papillomavirus (HR-HPV) infections, and the results of liquid-based cytology using either the Pearson chi-square test or Fisher’s exact test and the strength of associations of HR-HPV positivity and cytological abnormalities was quantified using odds ratios (Ors). Statistical significance was set at *p* < 0.05. Visualization of the settlement distribution of the HPV samples collected was accomplished through Canva (https://www.canva.com/, accessed on 15 July 2026). Figures were prepared for descriptive presentation only and were not used as a source of statistical inference.

## 3. Results

### 3.1. The Prevalence of HR-HPV Genotypes

**Figure 1:**
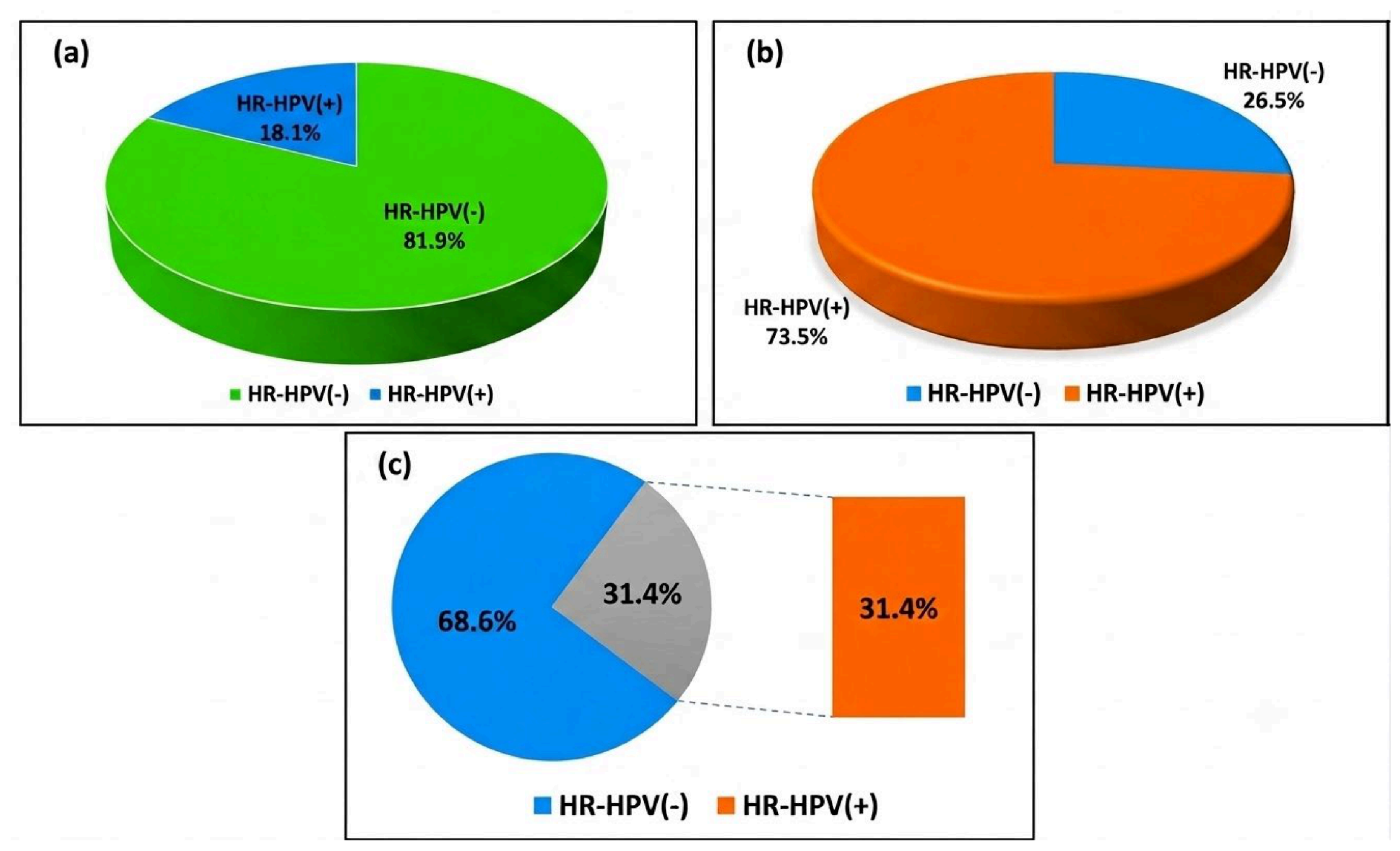
The prevalence of HR-HPV in the tested population.

The study included 1747 Bulgarian women aged 14–114 years. The mean age was 36.9 years (SD = 10.57), and the median age was 36.0 years. The maximum recorded age of 114 years was verified against the original clinical record. A sensitivity analysis excluding this extreme observation did not materially affect the overall age-related summary statistics or the main study findings. Because of the retrospective nature of the dataset, detailed clinical information regarding the indication for testing was not available. The majority of outpatients (76.0%; 95% CI: 73.9% to 78.0%, *n* = 1328) underwent routine HPV PCR prophylactic testing, while 24.0% (95% CI: 22.0% to 26.1%, *n* = 419) presented with clinical indications for HPV testing. Among women undergoing routine testing, the prevalence of HR-HPV was 18.1% (95% CI: 16.0% to 20.3%, *n* = 240) (Figure 1a). Among individuals with clinical reasons for undergoing HPV testing, the proportion was elevated at 73.5% (95% CI: 69.0% to 77.7%, *n* = 308) (χ^2^ = 454.6, *p* < 0.00001) (Figure 1b). This category includes not only women presenting with symptoms but also those referred due to prior abnormal findings from HPV PCR tests, cytology, or visual examinations. This suggests a preselected referral group with a significant probability of HPV infection or cervical abnormalities prior to testing. In the trial group as a whole, 548 women were found to have HR-HPV infection, resulting in a total HR-HPV infection rate of 31.4% (95% CI: 29.2% to 33.6%, *n* = 548) (Figure 1c).

### 3.2. The Distribution of the High-Risk HPV Genotypes Within the Studied Cohort

**Figure 2:**
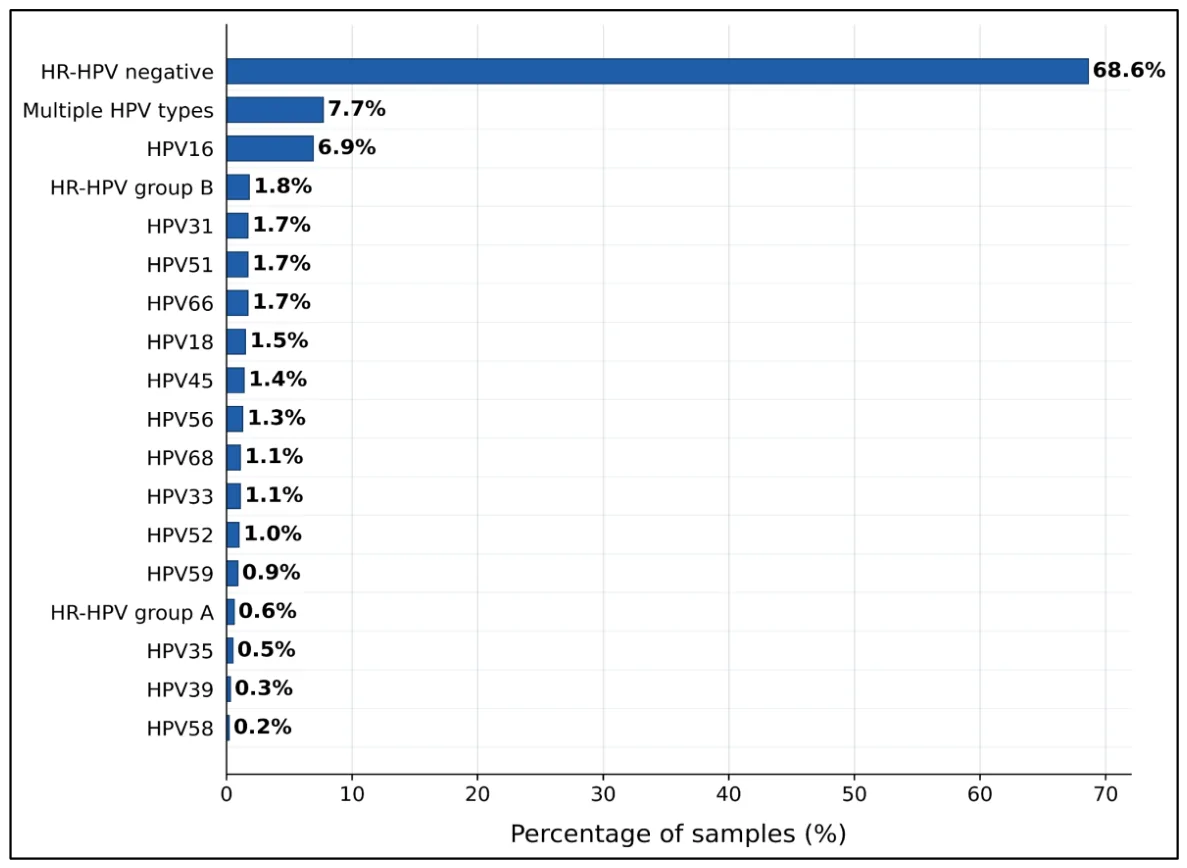
Comprehensive Overview of High-Risk HPV Genotypes Distribution Within the Examined Cohort.

The prevalence of HR-HPV among the entire tested group was recorded as follows: HPV16 was present in 6.9% (95% CI: 5.7% to 8.2%, *n* = 120); HPV18 in 1.5% (95% CI: 1.0% to 2.3%, *n* = 27); HPV31 in 1.7% (95% CI: 1.2% to 3.4%, *n* = 30); HPV33 in 1.1% (95% CI: 0.6% to 1.7%, *n* = 19); HPV35 in 0.5% (95% CI: 0.2% to 0.9%, *n* = 9); HPV39 in 0.3% (95% CI: 0.1% to 0.7%, *n* = 6); HPV45 in 1.4% (95% CI: 0.8% to 2.0%, *n* = 24); HPV51 in 1.7% (95% CI: 1.1% to 2.4%, *n* = 29); HPV52 in 1.0% (95% CI: 0.6% to 1.6%, *n* = 18); HPV56 in 1.3% (95% CI: 0.8% to 1.9%, *n* = 22); HPV58 in 0.2% (95% CI: 0.04% to 0.5%, *n* = 3); HPV59 in 0.9% (95% CI: 0.5% to 1.5%, *n* = 16); HPV66 in 1.7% (95% CI: 1.1% to 2.4%, *n* = 29); and HPV68 in 1.1% (95% CI: 0.6% to 1.7%, *n* = 20) (Figure 2).

Among all the women tested, 7.7% (95% CI: 6.5–9.1%, *n* = 135) were found to have infections with multiple HPV genotypes. Those who tested positive for one or more HPV genotypes from HR-HPV group A and HR-HPV group B were recorded at 0.6% (95% CI: 0.2 to 1.0%, *n* = 10) and 1.8% (95% CI: 1.2 to 2.5%, *n* = 31), respectively.

**Figure 3:**
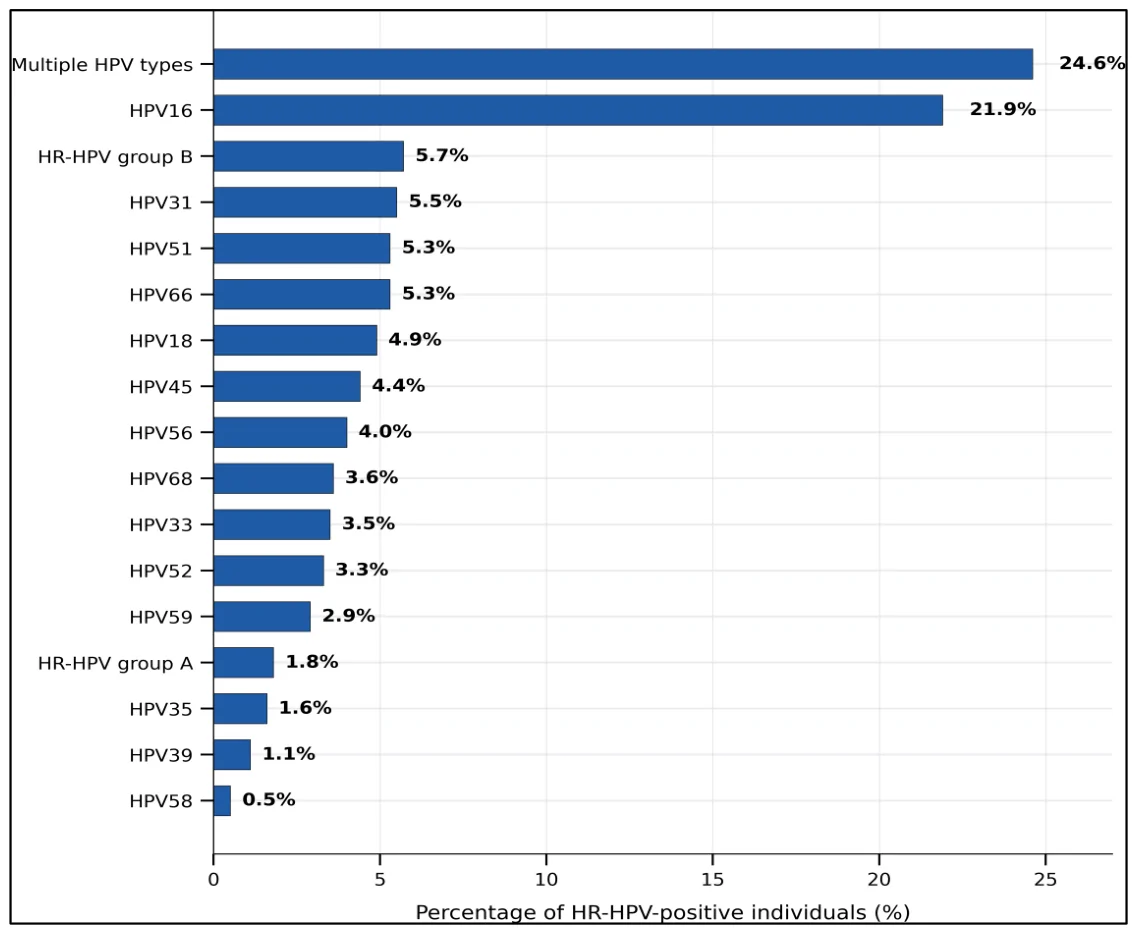
HR-HPV genotype prevalence in the studied HPV-positive group.

The most commonly detected HR-HPV genotypes among positive cases were HPV16- 21.9% (95% CI: 18.5% to 25.6%, *n* = 120), HPV31- 5.5% (95% CI: 3.7% to 7.7%, *n* = 30), HPV51- 5.3% (95% CI: 3.6% to 7.5%, *n* = 29), HPV66- 5.3% (95% CI: 3.6% to 7.5%, *n* = 29), HPV18- 4.9% (95% CI: 3.3% to 7.1%, *n* = 27), HPV45- 4.4% (95% CI: 2.8% to 6.5%, *n* = 24), HPV56- 4.0% (95% CI: 2.5% to 6.0%, *n* = 22), HPV68- 3.6% (95% CI: 2.3% to 5.6%, *n* = 20) and HPV33- 3.5% (95% CI: 2.1% to 5.4%, *n* = 19) (Figure 3).

Among women with multiple HR-HPV infections, infection with two HR-HPV genotypes was the most common pattern, occurring in 72.6% (95% CI: 64.3–79.9%; *n* = 98) of cases. Infection with three HR-HPV genotypes was observed in 20.7% (95% CI: 14.3–28.6%; *n* = 28), while four HR-HPV genotypes were detected in 5.2% (95% CI: 2.1–10.4%; *n* = 7). One woman each had infection with six and seven HR-HPV genotypes, representing 0.7% (95% CI: 0.2–4.1%; *n* = 1) for each category. Among the women evaluated according to the Group A and Group B HR-HPV categories, 7.5% (95% CI: 5.4–10.0%; *n* = 41) had HR-HPV infections, including both single and multiple-genotype infections. Among women with multiple HR-HPV infections, HPV16 was detected in 51.8% (95% CI: 43.1–60.5%; *n* = 70), indicating that HPV16 frequently occurred as part of a multiple-genotype infection.

**Figure 4:**
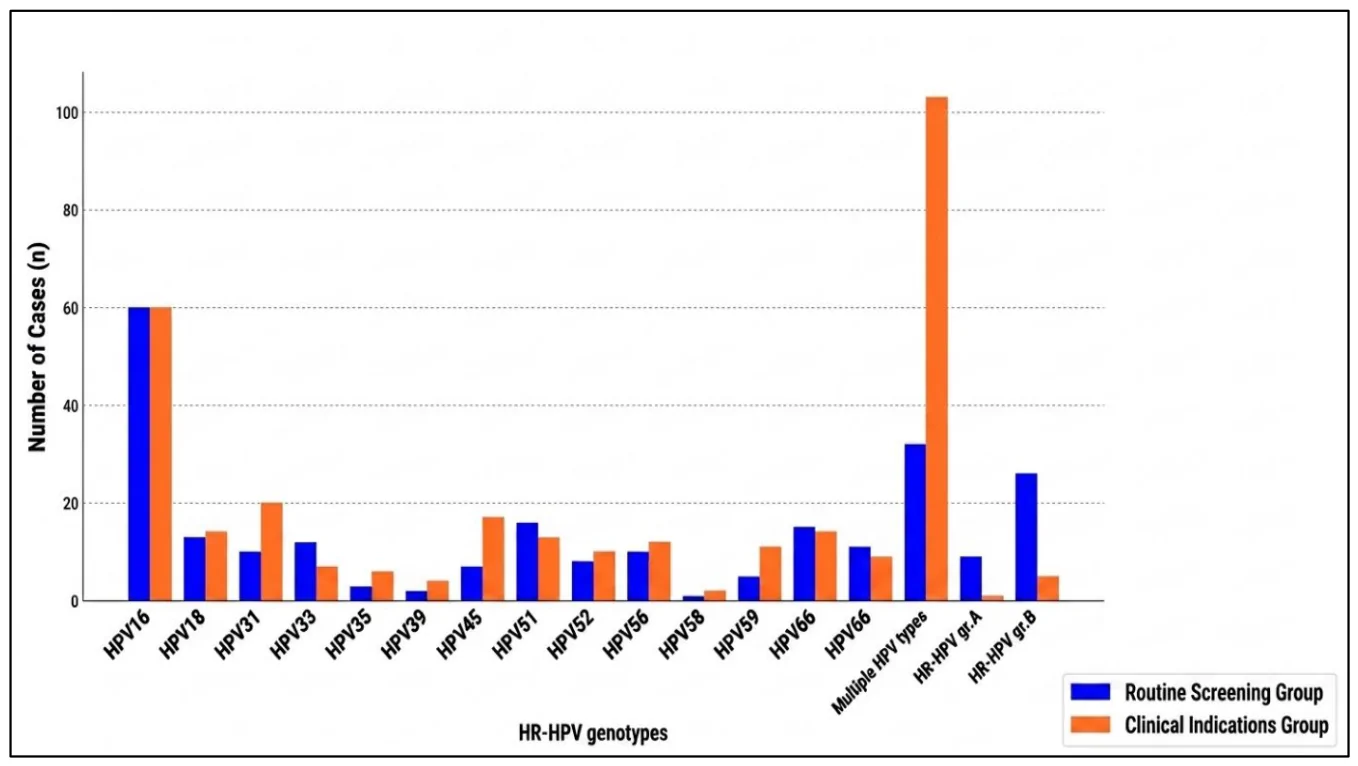
HR-HPV distribution in routinely screened group vs. group with clinical indications.

HPV16 emerged as the predominant strain among positive samples with a single genotype in both categories of women: those undergoing routine HPV screening and those tested due to clinical reasons. Of these cases, half (50%, 95% CI: 40.7% to 59.3%, *n* = 60) were from women participating in HPV screening, while the other half (50%, 95% CI: 40.7% to 59.3%, *n* = 60) were from women tested based on clinical indications (Figure 4).

The detection rate of HPV16 was 4.5% (95% CI: 3.5% to 5.8%, *n* = 60) in the routine screening group, compared to 14.3% (95% CI: 11.1% to 18.0%, *n* = 60) in the clinically indicated group, highlighting a notably higher detection rate in the latter. We assessed the proportion of HPV16 among women who tested positive for any high-risk HPV type within each group. Among HR-HPV positive women, HPV16 accounted for 25.0% (95% CI: 19.6% to 30.1%, *n* = 60) in the routine screening group and 19.5% (95% CI: 15.2% to 24.3%, *n* = 60) in the clinically indicated group.

Among women tested for clinical indications the HR-HPV genotypes that were most frequently observed included HPV 45- 70.8% (95% CI: 48.9% to 80.4%, *n* = 17), HPV 59-68.7% (95% CI: 41.3% to 88.9%, *n* = 11), HPV 31–66.7% (95% CI: 47.2% to 82.7%, *n* = 20), HPV 35–66.7% (95% CI: 29.9% to 92.5%, *n* = 9), HPV 52–55.6% (95% CI: 30.7% to 78.5%, *n* = 10), HPV 56–54.5% (95% CI: 32.2% to 75.6%, *n* = 12), and HPV 18–51.8% (95% CI: 31.9% to 71.3%, *n* = 14). Among the women who were tested for clinical reasons, 76.3% (95% CI: 68.2% to 83.2%, *n* = 103) were found to have infections involving multiple genotypes. The observed higher frequency of multiple HR-HPV infections in women tested for clinical indications may be associated with HPV persistence and cervical abnormalities. Nevertheless, the study design does not permit causal inference, and no conclusions can be drawn regarding a direct contribution of HPV infection or specific genotype combinations to the clinical symptoms observed.

### 3.3. The Prevalence of HR-HPV Genotypes in the Defined Age Groups

The tested women were categorized in thirteen age groups (14–20, 21–25, 26–30, 31–35, 36–40, 41–45, 46–50, 51–55, 56–60, 61–65, 66–70, 71–75, >75). The distribution of HR-HPV genotypes across age groups among HR-HPV-positive women (*n* = 548) is shown in Table 1.

The incidence of HR-HPV infections exhibited notable variation across different age groups. The prevalence of genotypes was most pronounced among young adults, with the 26–30 age group experiencing the highest overall impact at 20.1% (95% CI: 16.8% to 23.7%, *n* = 110). This was closely followed by the 31–35 age group at 18.2% (95% CI: 15.1% to 21.7%, *n* = 100) and the 36–40 age group at 15.9% (95% CI: 12.9% to 19.2%, *n* = 87). Collectively, individuals aged 21 to 40 years constituted more than two-thirds—68.6% (95% CI: 64.5% to 72.5%, *n* = 376)—of all positive cases. It was anticipated that most women in this age groups would clear the HPV infection within 1–2 years, as they are generally immunocompetent. In contrast, the detection of HR-HPV significantly diminished in older age groups, with those aged 51 years or older comprising only 6.2% (95% CI: 4.3% to 8.6%, *n* = 34) of the total sample; however, the small number of women in these strata resulted in relatively imprecise estimates, and this pattern should therefore be interpreted cautiously. While most genotypes dropped precipitously after age 40, HPV51 demonstrated a unique bimodal distribution with a secondary peak re-emerging in the 41–50 age group. There were no HR-HPV positive women in the group above 75 years old. To assess whether the distribution of high-risk human papillomavirus genotypes varied significantly across age demographics, a Pearson chi-square test of independence was performed. The results revealed a highly statistically significant association between patient age and the specific HR-HPV genotype profile (χ^2^ = 240.5; *p* < 0.001). This variation was primarily attributed to a marked concentration of high-burden strains—particularly HPV16 and multiple-type co-infections—among young adults aged 21 to 40 years, followed by a sharp, statistically distinct decline in overall viral diversity and case numbers in groups older than 50 year.

### 3.4. Settlement Distribution of the HPV Samples

**Figure 5:**
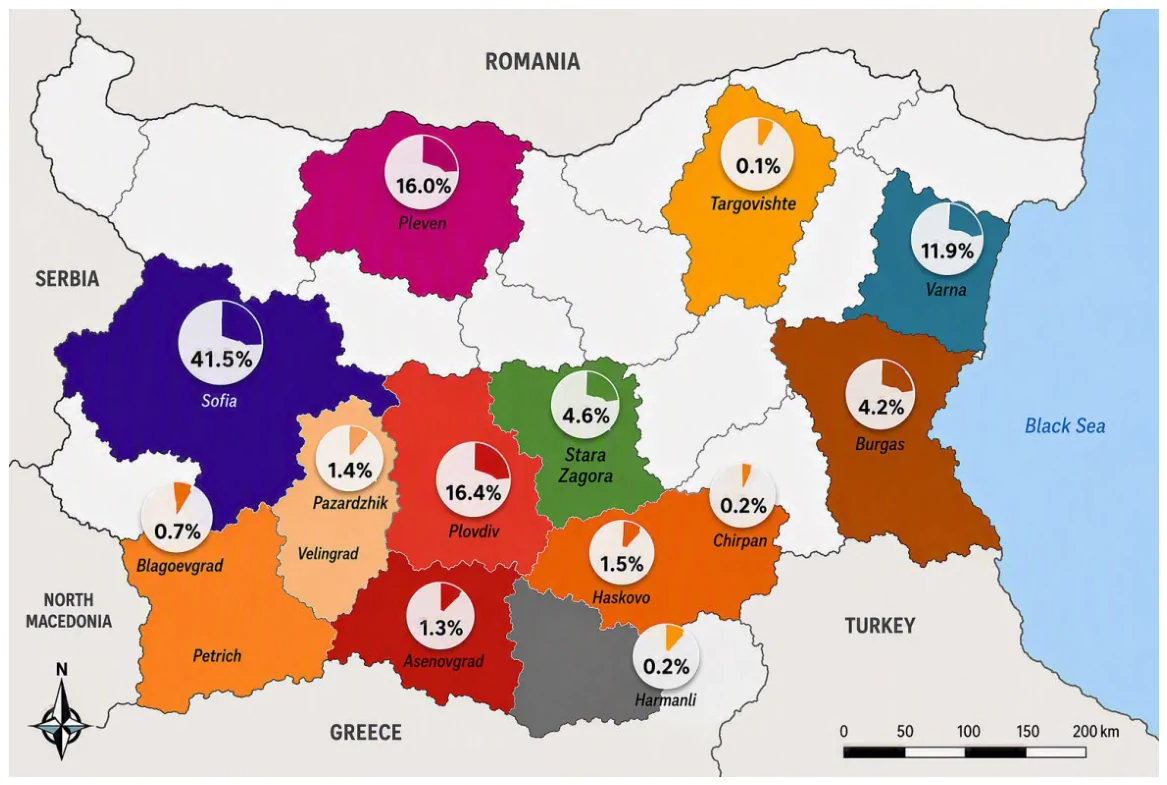
HPV samples obtained across different regions in Bulgaria.

Cervical swab samples were collected across 13 distinct geographic regions of Bulgaria for this study. This approach of collecting samples from various locations throughout Bulgaria aimed to improve the study’s representativeness and applicability. The primary core tier comprised Sofia with 41.5% (95% CI: 39.2 to 43.8%, *n* = 725); Plovdiv with 16.4% (95% CI: 14.6 to 18.2%, *n* = 286); Pleven with 16.0% (95% CI: 14.3 to 17.8%, *n* = 280); and Varna with 11.9% (95% CI: 10.5 to 13.6%, *n* = 209). These cities accounted for the vast majority—85.8% (95% CI: 84.1 to 87.5%, *n* = 1500)—of the total HPV sample registry (Figure 5).

An intermediate tier of regional secondary administrative centers provided moderate sample volumes, led by Stara Zagora with 4.6% (95% CI: 3.6 to 5.6%, *n* = 80) and Burgas with 4.2% (95% CI: 3.3 to 5.3%, *n* = 74), followed by Haskovo with 1.5% (95% CI: 0.9 to 2.2%, *n* = 26), Pazardzhik with 1.4% (95% CI: 0.8 to 2.0%, *n* = 24), and Asenovgrad with 1.3% (95% CI: 0.8 to 1.9%, *n* = 22). Conversely, peripheral and smaller municipal cohorts demonstrated limited representation, with Blagoevgrad contributing 0.7% (95% CI: 0.4 to 1.2%, *n* = 12), Harmanli 0.2% (95% CI: 0.06 to 0.6%, *n* = 4), Chirpan 0.2% (95% CI: 0.04 to 0.5%, *n* = 3), and Targovishte 0.1% (95% CI: 0.01 to 0.4%, *n* = 2), cumulatively contributing just 1.2% (95% CI: 0.7 to 1.8%, *n* = 21) of the data pool.

### 3.5. The Prevalence of HR-HPV Genotypes and LBC Results

In 64.9% of the LBC samples (95% CI: 62.6 to 67.1%, *n* = 1134), a cytological examination was performed. Of these, 76.8% (95% CI: 74.2 to 79.2%, *n* = 871) were analyzed using the SurePath technique, while the remaining 23.2% (95% CI: 20.7 to 25.7%, *n* = 263) were processed using ThinPrep PreservCyt liquid-based cytology. Pathology specialists assessed the LBC samples using the 2014 Bethesda System (Table 2).

The examination of liquid-based cytology results in relation to specific high-risk human papillomavirus genotypes uncovers distinct patterns in infection rates and their association with cervical tissue abnormalities. Among women included in the paired HPV–cytology analysis, 45.3% (95% CI: 42.3 to 48.2%, *n* = 513) were HR-HPV positive, compared with 34.9% (95% CI: 31.1 to 38.8%, *n* = 214) among women without available cytology results. This difference was statistically significant (χ^2^ = 17.76, *p* < 0.001), indicating that HR-HPV positivity was more frequent among women included in the paired analysis. Additionally, 63.1% (95% CI: 58.2 to 67.6%, *n* = 264) of women in the paired HPV–cytology analysis were tested due to clinical indications, compared with 36.9% (95% CI: 32.4 to 41.2%, *n* = 155) among women without available cytology results. This difference was statistically significant (χ^2^ = 104.9, *p* < 0.001), indicating that the clinical indication for testing differed substantially between women included in and excluded from the paired analysis. Among the paired cytology subset (*n* = 1134; 64.9% of the total cohort), the majority presented with normal cytology- 75.3% (95% CI: 72.7 to 77.7%, *n* = 854) and were classified as negative for intraepithelial lesion or malignancy (NILM). The remaining 24.7% (95% CI: 22.2 to 27.3%, *n* = 280) exhibited abnormal cytology findings. Atypical squamous cells of undetermined significance (ASC-US) were found in 14.7% (95% CI: 12.7 to 16.9%, *n* = 167), and low-grade squamous intraepithelial lesions (LSIL) were present in 6.9% (95% CI: 5.5 to 8.6%, *n* = 79). High-grade squamous intraepithelial lesions (HSIL) were less common, occurring in 3.0% (95% CI: 2.1 to 4.2%, *n* = 34) of the women with cytology results. HPV16 emerged as the predominant genotype in both the ASC-US group at 19.4% (95% CI: 10.4 to 31.4%, *n* = 12) and the LSIL group at 15.4% (95% CI: 4.4 to 34.8%, *n* = 4), excluding cases with multiple HR-HPV genotype infections.

Among those with multiple genotype infections, most premalignant lesions were identified: ASC-US was present in 30.6% (95% CI: 19.6 to 43.6%, *n* = 19), LSIL in 34.6% (95% CI: 17.2 to 55.6%, *n* = 9), and HSIL in 7.7% (95% CI: 0.02 to 36.0%, *n* = 1). Notably, a significant portion of HSIL cases, 61.8% (95% CI: 43.6 to 77.8%, *n* = 21), were found in patients who tested negative for HR-HPV. A crucial insight for clinical screening is the rate of latent or cytologically silent viral shedding observed in the normal cohort. Among the patients classified as NILM (*n* = 854), 27.3% (95% CI: 24.3 to 30.4%, *n* = 233) tested positive for at least one high-risk HPV strain despite having normal cell morphology.

Within the subgroup of cytologically normal but HR-HPV-positive women (NILM/HR-HPV-positive; *n* = 233), HPV16 was the most frequently detected genotype, accounting for 26.2% (61/233) of infections. Multiple HR-HPV infections were observed in 21.0% (49/233), followed by HR-HPV group B in 13.3% (31/233), while HPV18 and HPV31 were each detected in 5.6% (13/233). Among women with abnormal cytology (*n* = 280), HR-HPV positivity was observed in 36.1% (101/280). Abnormal cytology was associated with higher odds of HR-HPV positivity compared with NILM cytology (OR = 1.50; 95% CI: 1.13 to 2.00%; χ^2^ = 7.84, *p* = 0.0051). Among women with paired HPV-cytology results who underwent HPV testing for clinical indications (*n* = 264), NILM was observed in 56.4% (149/264), ASC-US in 25.8% (68/264), LSIL in 12.5% (33/264), and HSIL in 5.3% (14/264).

## 4. Discussion

This retrospective study provides important data on the prevalence and genotype distribution of HR-HPV infection in a Bulgarian cohort of 1747 women. The mean (36.9 years) and median (36.0 years) age of this cohort mirror standard European cervical screening populations, which typically capture women during their peak reproductive and post-reproductive years [12]. However, the inclusion of a 114-year-old participant represents an extreme statistical outlier that expands the lifetime epidemiological window significantly beyond standard cohorts. The 3:1 ratio between routine screening and diagnostic indication subgroups represents a robust distribution. In Western Europe, countries operating under centralized, organized invitation systems (the Netherlands, Scandinavia) often see routine screening shares exceed 85–90%. The higher rate of symptomatic/clinically driven presentations (24.0%) in this Bulgarian cohort is highly characteristic of Central and Eastern European healthcare structures, where opportunistic, patient- or provider-initiated testing remains more prominent [13].

According to large-scale global meta-analyses published by *The Lancet Microbe*, HR-HPV prevalence among women with normal cytology ranges between 4.1% and 6.0% in Western Europe, and averages roughly 11.7% globally [14]. The epidemiological landscape of human papillomavirus in South-Eastern Europe (the Balkan region, including Bulgaria, Romania, Serbia, Greece, and Albania) is marked by a significantly higher cervical HPV prevalence and cervical cancer burden compared to Western and Northern Europe. Some studies documented the prevalence of HPV in Eastern Europe at 21.4%, while in Western Europe, it stands at 9.0%, with Spain reporting a low of 2% and France and Belgium around 12% [15]. In Poland, the rate is 14.4%, the Czech Republic shows 25.6%, and Lithuania records 24.2% [16]. In Greece, HPV prevalence varies between 2.5% and 50.7% [12]. Research from Turkey indicates HR-HPV prevalence ranging from 2.4% to 47.7% [17]. In Italy, the HPV-DNA positivity rate among women was found to be 35.9% [18]. A meta-analysis determined that HPV DNA prevalence in women with normal cytology varied from 5.3% to 35.6% [19]. The differences in reported HPV prevalence may be attributed to variations in age distribution, sampling techniques, screening history, and vaccination status of the studied populations. According to a few studies, in Bulgaria, the prevalence of high-risk human papillomavirus infection is significant, with rates among women with normal cytology ranging from about 29.8% to 38.8%, as shown in extensive population-based studies [7,20,21]. In our research, HR-HPV prevalence within a routine asymptomatic screening cohort was detected at 18.1%, substantially higher than figures observed across broader European populations. However, our data aligned precisely with the elevated boundaries observed in Eastern Europe, where background viral pools historically hover near 14.0–18.0% [22]. This disparity is driven by historically lower national vaccination coverage and slower transitions to primary molecular HPV screening setups relative to Western counterparts.

The overall HR-HPV prevalence was reported at 31.4%, with a distinct difference between women undergoing routine prophylactic testing and those tested for clinical reasons. In the group where clinical indications were present, a high positivity rate for HR-HPV was noted at 73.5%. It is essential to acknowledge that when evaluating diagnostic accuracy, it is important to compare the results of the index test with a reference standard and compute key performance metrics, including sensitivity, specificity, positive predictive value, and negative predictive value. Nevertheless, due to constraints in the available reference standard data, such analyses could not be conducted in this study. We also recognize the possibility of selection bias, referral bias, and indication bias within our study design and population. These biases might have contributed to the observed elevated HR-HPV positivity rate, leading to estimates that exceed those anticipated in a general screening or symptomatic population.

Globally, HPV16 reigns uniformly as the most dominant oncogenic type, bound to approximately 20–25% of positive screening samples and upwards of 50–65% of severe precancers (cervical intraepithelial neoplasia (CIN)3+ or cancers [23]. The Bulgarian dataset reinforced this paradigm perfectly. The genotype profile was dominated by HPV16, which was the most frequently detected genotype both in the overall cohort and among HR HPV-positive women, which underscored its status as the primary oncogenic driver within the target population, irrespective of clinical presentation. HPV16 represented 21.9% of positive cases, reinforcing its central role as the leading oncogenic genotype in this Bulgarian cohort. This finding is consistent with the broader literature identifying HPV16 as the most important high-risk genotype involved in cervical carcinogenesis and high-grade precursor lesions [24]. Interestingly, the absolute equilibrium mentioned in the manuscript, in single-genotype HPV16 distribution—equally divided at 50% between the asymptomatic screening cohort and the symptomatic clinical indication group—suggests that while HPV16 possesses high oncogenic potential, its initial detection pattern remains uniform across different entry points in the healthcare system. It is important to note that the absolute number of HPV16-positive cases does not equate to a balanced distribution between the groups. Instead, detection rates and proportions relative to denominators provide a more accurate reflection of the differences in HPV16 prevalence and distribution between women screened routinely and those screened for clinical reasons. This refined analysis supports the understanding that HPV16 prevalence varies based on the population screened and clinical context, which is essential for gaining accurate epidemiological insights and effectively tailoring screening strategies. This also highlights the dangerous clinical nature of HPV 16 genotype as it circulates just as silently among healthy, asymptomatic screening outpatients as it does within heavily symptomatic, clinical referral populations [25].

Crucially, the positioning of HPV31, HPV51, and HPV66 ahead of HPV18 reveals a distinct geographic variance. In broad global meta-analyses, HPV18 typically cements itself securely as the second most common genotype globally [26]. In our cohort, however, it dropped to 5th place (4.9%). The inflation of non HPV16/HPV18 HR-HPV genotypes, including HPV31, HPV51, HPV66, HPV45, HPV56, HPV68, and HPV33, highlights the epidemiological footprint of the region, emphasizing the importance of extended genotyping beyond HPV16 and HPV18 alone. Conversely, the data from the colposcopy cohort reveals a distinct topological shift in viral architecture. The high relative frequencies of HPV 45 (70.8%), HPV 59 (68.7%) and HPV 31 (66.7%) indicated that these specific genotypes are heavily enriched in patients exhibiting visible cervical pathology. This strong clustering among colposcopy referrals highlights the clinical importance of non-HPV16 high-risk strains, which might be underestimated in broader population screenings but clearly present a significant risk for neoplastic progression.

Investigating the prevalence of HR-HPV, the distribution of HPV genotypes, and their associations with cytological abnormalities among women undergoing routine screening or presenting with gynecological symptoms provides important insights into patterns relevant to cervical cancer risk assessment. However, associations identified in univariable analyses may be influenced by potential confounding factors and therefore should not be interpreted as independent associations unless they remain statistically significant after adjustment for relevant covariates. Consistent with the broader evidence, our findings indicate that HR-HPV infection, particularly with genotypes such as HPV16, HPV52, and HPV58, is associated with an increased likelihood of cytological abnormalities. These findings support the potential value of HPV genotyping as an adjunct to cytology for refining individualized risk assessment and informing cervical cancer screening strategies. Such an approach may facilitate more efficient identification of women at increased risk and contribute to improved cervical cancer prevention [27].

In the genotype distribution, multiple HR-HPV infections, such as dual, triple, or quadruple, were a significant clinical factor [28]. The presence of multiple infections may influence genotype-specific prevalence estimates and cytological associations, and genotype-specific associations should therefore be interpreted with appropriate caution. Among 135 women, more than one HR-HPV genotype was identified, with HPV16 appearing in over half of these multiple-infection instances. This finding indicates that HPV16 often coexists with other high-risk genotypes within the population studied. Women who tested positive for one or more genotypes from HR-HPV group A and HR-HPV group B were categorized separately. Notably, one woman was infected with 6 and another one with 7 HR-HPV genotypes. Within the subpopulation of women harboring multi-infections, the rapid descent from dual (72.6%) to hyper-infections (6–7 strains, 1.4%) aligns with standard competitive exclusion principles in virology. It is rare for a host’s cervical microenvironment to sustain more than three high-risk viral strains simultaneously without intense immune signaling or severe underlying epithelial disruption [29]. The observation that 76.3% of multi-infections resided within the diagnostic/clinical group provides vital clinical context. Modern cross-sectional trials indicate that multi-genotype carriage significantly decreases a woman’s cumulative viral clearance rate. The simultaneous presence of multiple strains hampers cellular immune response mechanisms, driving the persistence of abnormalities and the emergence of cytological lesions [30]. Furthermore, HPV16 presence in 51.8% of these multi-type profiles is critical. In academic literature, it remains heavily debated whether multiple infections act synergistically to cause cancer, or if a single dominant strain drives the lesion while others act as innocent bystanders. The fact that HPV16 acts as the core anchor in over half of these complex multi-infections suggests it leverages multi-strain environments to maintain long-term persistence [31]. While interpreting multiple infections clinically is challenging, their occurrence might indicate recent exposure, ongoing infection, or intersecting transmission networks. In screening, these multiple infections can complicate risk assessment based on genotypes, especially when tests vary in reporting individual genotypes versus grouped HR- HPV categories [32].

The age distribution provides further insight into the epidemiology of HR-HPV infection within the studied cohort, reflecting typical global behavioral patterns associated with peak sexual activity and the acquisition of new viruses following sexual initiation [33]. HR-HPV positivity was predominantly observed in young adults, particularly among women aged 21–40 years, who constituted over two-thirds (68.6%) of all positive cases. This trend is consistent with the established natural history of HPV infection, where acquisition is more prevalent at younger ages, and many infections are subsequently cleared. The significant decline in positivity after age 40, reaching a low of 6.2% in women over 50, suggests normal and effective viral clearance. In healthy, immunocompetent individuals, more than 90% of new infections are naturally resolved through cell-mediated immunity within 12 to 24 months. While young adults exhibit a broad and varied range of high-burden strains and multiple infections, older groups demonstrate a reduced and less diverse viral presence [34]. The identification of a bimodal distribution for HPV51, reappearing between ages 41–50, represents a notable academic finding. Globally, bimodal patterns are frequently observed for overall HPV presence, typically showing an initial peak in the 20s and a secondary rise during perimenopausal or postmenopausal phases (ages 45–55) [35]. This secondary increase is generally attributed to two competing biological theories: hormonal changes during perimenopause that alter the cervical epithelium, facilitating the reactivation of latent infections acquired earlier, and changes in partner dynamics and exposure to new viral strains later in life. The distinctive aspect of this dataset is that the bimodal resurgence is specifically associated with HPV51 rather than a general resurgence of all strains. This suggests that HPV51 may possess unique latency or reactivation characteristics within the Bulgarian population, warranting closer clinical monitoring. The moderate association between age and genotype profile in the Bulgarian cohort indicates that age should remain a crucial factor in interpreting screening results and making follow-up decisions [36].

From a methodological standpoint, sampling from 13 distinct administrative districts effectively reduces the risk of selection biases inherent in single-center studies, thereby ensuring that this research is broadly representative of urban areas in Bulgaria. Geographically, the sample registry included the majority of samples originating from Sofia, Plovdiv, Pleven, and Varna. These four cities accounted for 85.8% of the total samples. The concentration of data in these major urban centers underscores the logistical constraints of Bulgaria’s clinical infrastructure. Sofia, Plovdiv, Pleven, and Varna serve as the primary hubs for specialized diagnostic molecular pathology and centralized testing laboratories. Consequently, while the dataset is valuable and geographically diverse, it may not fully represent the situation in smaller municipalities (1.2%) or rural areas. In rural or peripheral regions, access to primary liquid-based cytology and PCR testing is a logistical limitation, resulting in an underestimation of the actual viral burden in these areas due to lower local screening rates. Future studies with more balanced regional recruitment could help ascertain whether HR-HPV prevalence and genotype distribution differ between urban, semi-urban, and peripheral populations.

The combined analysis of HR-HPV and cytology indicated that 75.3% of women with liquid-based cytology results were NILM, while 24.7% exhibited abnormal cytology. ASC-US was the most prevalent abnormality, followed by LSIL and HSIL. A moderate association was observed between HR-HPV positivity and abnormal cytology, highlighting the biological connection between oncogenic HPV infection and epithelial transformation. Nevertheless, the relationship between HR-HPV status and cytological abnormality was not conclusive. The finding that 27.3% of NILM cases were HR-HPV positive is clinically significant. These women may represent early infections prior to detectable cytological changes, transient infections that could resolve spontaneously, or persistent infections requiring monitoring. This emphasizes the importance of co-testing and risk-based follow-up, particularly when HPV16 or multiple HR-HPV genotypes are identified [37]. HPV testing alone can identify women at risk before cytological abnormalities appear, while cytology assists in identifying women with current morphological evidence of epithelial abnormality. This balance is essential in modern cervical screening, as HR HPV testing is sensitive but may lack specificity without triage [38]. The observation that a significant portion of HSIL cases were HR-HPV negative warrants careful consideration. In this dataset, 61.8% of HSIL cases were reported among women who tested negative for HR-HPV. The detection of high-grade squamous intraepithelial lesions in individuals testing negative for high-risk human papillomavirus represents a critical clinical paradox, primarily driven by false-negative viral assays or distinct biological phenomena, and should be interpreted cautiously. Formally published data, including studies in the Journal of Medical Virology and Histopathology, demonstrate that a meaningful subset of patients with high-grade dysplasia initially return a negative HR-HPV result. Confirmatory testing on an alternative platform was not systematically performed, and false-negative molecular results, sampling issues, limited genotype coverage, or diagnostic misclassification cannot be excluded. Future studies should include quality-assurance procedures for apparently HPV-negative high-grade lesions [39,40]. Mechanistically, this discordance frequently stems from viral integration into the host genome, which often involves the deletion or disruption of the HPV L1 gene- the primary target for most commercial DNA assays. Consequently, while oncogenic E6 and E7 expression continues to drive cellular atypia, the virus becomes undetectable by standard molecular screening. This highlights the vital role of concurrent cytological evaluation and immunohistochemical markers like p16 to ensure high-grade lesions are not missed [41]. Therefore, these HSIL/HR-HPV-negative cases should be prioritized for follow-up, ideally through repeat HPV testing, expert cytology review, colposcopy, and histopathological correlation where possible [42].

A significant methodological strength of this study is the implementation of liquid-based cytology and molecular HPV testing within a real-world laboratory context. The study employed both ThinPrep and SurePath-based workflows, with cytology specimens evaluated in accordance with the 2014 Bethesda System [43]. Additionally, two molecular platforms were utilized: Seegene Anyplex II HPV28 and Abbott Alinity m HR HPV. This approach mirrors routine diagnostic practice but also presents a notable limitation, as the assays possess partially overlapping yet distinct genotype panels and differing reporting formats. Numerous studies have reported substantial concordance between these assays in detecting HPV positivity and specific genotypes, as evidenced by Cohen’s kappa statistics indicating good to excellent agreement (e.g., κ values generally >0.6) [44]. Nevertheless, minor variations in sensitivity and the detection of certain genotypes necessitate careful interpretation. For a valid pooled analysis, it is essential to separate HPV result groups based on the distinct usage, genotyping capabilities, and reporting styles of the two molecular platforms. Harmonization can be achieved by maintaining common genotypes, employing assay-specific group categories as reported, and acknowledging multiple infections, thereby accommodating platform differences while preserving biological and epidemiological significance across datasets [45].

This retrospective cross-sectional study has several important limitations that should be considered when interpreting the findings. First, a substantial proportion of the overall cohort (35.1%, *n* = 613) lacked paired cytology results. Furthermore, women undergoing routine screening and those tested for clinical indications may differ in underlying demographic, behavioural, and clinical characteristics beyond the indication for testing itself, introducing the potential for selection bias. The limited availability of information on HPV vaccination status, prior screening participation, previous cytological and HPV results, treatment history, sexual behaviour, smoking status, immunosuppressive conditions, hormonal contraceptive use, and other established epidemiological risk factors restricted our ability to fully account for potential confounding. These factors may have influenced both the observed genotype distribution and age-specific prevalence patterns.

In particular, differential HPV vaccination uptake across birth cohorts could reduce the prevalence of vaccine-targeted genotypes, especially HPV16 and HPV18, among younger women, potentially altering the relative distribution of non-vaccine genotypes. Similarly, variations in screening participation between age groups may affect genotype-specific prevalence estimates, as women undergoing regular screening are more likely to have precancerous lesions detected and managed earlier, whereas under-screened individuals may be more likely to harbour persistent infections. Differences in sexual behaviour, including age at sexual debut and number of sexual partners, may further influence HPV acquisition and transmission dynamics and could partly account for age-related variations in infection prevalence. Smoking and immunosuppressive conditions are known to impair viral clearance and increase the likelihood of persistent and multiple HPV infections, potentially affecting both overall prevalence and genotype-specific frequencies. In addition, hormonal contraceptive use has been associated with changes in cervical epithelial susceptibility and HPV persistence, which may contribute to differences observed across reproductive age groups. Because many of these factors vary between generations and age strata, reflecting evolving vaccination coverage, screening practices, healthcare access, and sexual health behaviours, residual confounding cannot be excluded. Consequently, the age-specific genotype distributions observed in this study should be interpreted with caution, as some of the differences may reflect unmeasured behavioural, preventive, and healthcare-related factors rather than solely underlying biological patterns of HPV circulation. Future prospective studies incorporating comprehensive epidemiological and clinical data are warranted to better delineate the independent determinants of HPV genotype distribution and age-specific prevalence.

The geographic distribution of the study population was predominantly concentrated in major cities, and the obtained samples were mainly the result of the distribution of laboratory submissions which may limit the generalizability of the findings to the broader female population of Bulgaria. Furthermore, the cross-sectional design does not permit assessment of HPV persistence, spontaneous clearance, latency, reactivation, or progression of cytological abnormalities over time. Accordingly, the findings represent associations observed at the time of testing and cannot establish temporal or causal relationships. In particular, we cannot conclude that multiple HPV infections impede viral clearance, promote persistent infection, or cause cytological abnormalities. Similarly, the detection of HPV16 in multiple infections and the age-specific distribution of HPV51 should not be interpreted as evidence of long-term persistence, latency, or reactivation. These observations may generate hypotheses regarding potential genotype interactions and age-related patterns, but such hypotheses require confirmation in longitudinal studies involving repeated HPV testing and cytological follow-up of the same individuals.

Finally, some age-stratified subgroups, particularly among older women, included relatively few participants. Although 95% confidence intervals were calculated to reflect the uncertainty surrounding the estimates, the relatively wide intervals observed in these subgroups indicate limited precision. Consequently, genotype-specific patterns observed within small age strata should be interpreted cautiously and considered descriptive rather than definitive. Larger studies with adequate representation across age groups and longitudinal follow-up are needed to determine whether these observed patterns are reproducible and to clarify their clinical and epidemiological significance.

Despite these limitations, the study contributes valuable evidence on HR-HPV epidemiology in Bulgaria. The results show that HPV16 remains the dominant genotype, that non 16/18 genotypes and multiple infections are frequent, and that HR HPV positivity is significantly associated with abnormal liquid-based cytology. The detection of HR HPV infection among women with NILM cytology further supports screening models that integrate HPV genotyping and cytology rather than relying on either method alone. In clinical practice, these findings support risk-based triage strategies that consider HPV genotype, cytology category, age, and clinical context [46].

## 5. Conclusions

This study provides one of the most comprehensive contemporary assessments of high-risk human papillomavirus infection in a large Bulgarian female cohort and offers valuable insights into genotype distribution, age-specific prevalence patterns, and associations with cytological abnormalities. HR-HPV infection was common, with substantially higher positivity among women tested for clinical indications than among those undergoing routine screening. HPV16 remained the predominant genotype across the study population, confirming its central role in cervical carcinogenesis, while the high prevalence of non-16/18 HR-HPV genotypes, including HPV31, HPV51, HPV66, HPV45, and HPV56, highlights the considerable heterogeneity of HPV circulation in Bulgaria and underscores the importance of extended genotyping strategies beyond HPV16 and HPV18 alone.

The frequent detection of multiple HR-HPV infections and the observed age-related differences in genotype distribution suggest a complex epidemiological landscape that may have important implications for risk stratification and cervical cancer prevention. The significant association between HR-HPV positivity and abnormal cytology reinforces the biological link between oncogenic HPV infection and cervical epithelial transformation, whereas the detection of HR-HPV among women with negative cytology supports the value of incorporating molecular HPV testing into screening programs to identify women at increased risk before cytological abnormalities become apparent.

Collectively, these findings support the implementation of risk-based cervical cancer prevention strategies integrating HPV genotyping, cytology, age, and clinical context. They also provide important baseline epidemiological data for monitoring the future impact of HPV vaccination and screening policies in Bulgaria. Further large-scale prospective studies with longitudinal follow-up and comprehensive epidemiological characterization are warranted to clarify genotype-specific persistence patterns, evaluate the clinical significance of multiple infections, and better define the determinants of HPV-related disease progression in the Bulgarian population.

## Figures and Tables

**Table 1:** The overall prevalence of HR-HPV among the age groups.

HR-HPV Type	14–20	21–25	26–30	31–35	36–40	41–45	46–50	51–55	56–60	61–65	66–70	71–75	>75	Total *n* (%)
HPV16	3 (8.3)	12 (15.2)	25 (22.7)	21 (21.0)	25 (28.7)	20 (33.3)	7 (16.7)	2 (15.4)	1 (10.0)	3 (37.5)	1 (50.0)	0 (0.0)	0 (0.0)	120 (21.9)
HPV18	2 (5.6)	5 (6.3)	5 (4.5)	5 (5.0)	4 (4.6)	3 (5.0)	1 (2.4)	2 (15.4)	0 (0.0)	0 (0.0)	0 (0.0)	0 (0.0)	0 (0.0)	27 (4.9)
HPV31	2 (5.6)	5 (6.3)	3 (2.7)	3 (3.0)	6 (6.9)	0 (0.0)	7 (16.7)	3 (23.1)	0 (0.0)	0 (0.0)	1 (50.0)	0 (0.0)	0 (0.0)	30 (5.5)
HPV33	0 (0.0)	1 (1.3)	4 (3.6)	2 (2.0)	5 (5.7)	0 (0.0)	4 (9.5)	2 (15.4)	0 (0.0)	1 (12.5)	0 (0.0)	0 (0.0)	0 (0.0)	19 (3.5)
HPV35	0 (0.0)	1 (1.3)	1 (0.9)	3 (3.0)	3 (3.4)	1 (1.7)	0 (0.0)	0 (0.0)	0 (0.0)	0 (0.0)	0 (0.0)	0 (0.0)	0 (0.0)	9 (1.6)
HPV39	0 (0.0)	0 (0.0)	1 (0.9)	2 (2.0)	1 (1.1)	1 (1.7)	1 (2.4)	0 (0.0)	0 (0.0)	0 (0.0)	0 (0.0)	0 (0.0)	0 (0.0)	6 (1.1)
HPV45	1 (2.8)	1 (1.3)	2 (1.8)	6 (6.0)	8 (9.2)	2 (3.3)	1 (2.4)	1 (7.7)	2 (20.0)	0 (0.0)	0 (0.0)	0 (0.0)	0 (0.0)	24 (4.4)
HPV51	2 (5.6)	4 (5.1)	8 (7.3)	1 (1.0)	1 (1.1)	5 (8.3)	5 (11.9)	0 (0.0)	3 (30.0)	0 (0.0)	0 (0.0)	0 (0.0)	0 (0.0)	29 (5.3)
HPV52	0 (0.0)	1 (1.3)	5 (4.5)	3 (3.0)	2 (2.3)	4 (6.7)	0 (0.0)	1 (7.7)	0 (0.0)	2 (25.0)	0 (0.0)	0 (0.0)	0 (0.0)	18 (3.3)
HPV56	0 (0.0)	4 (5.1)	3 (2.7)	5 (5.0)	6 (6.9)	2 (3.3)	1 (2.4)	0 (0.0)	0 (0.0)	0 (0.0)	0 (0.0)	1 (100.0)	0 (0.0)	22 (4.0)
HPV58	1 (2.8)	0 (0.0)	0 (0.0)	2 (2.0)	0 (0.0)	0 (0.0)	0 (0.0)	0 (0.0)	0 (0.0)	0 (0.0)	0 (0.0)	0 (0.0)	0 (0.0)	3 (0.5)
HPV59	2 (5.6)	2 (2.5)	4 (3.6)	4 (4.0)	3 (3.4)	1 (1.7)	0 (0.0)	0 (0.0)	0 (0.0)	0 (0.0)	0 (0.0)	0 (0.0)	0 (0.0)	16 (2.9)
HPV66	3 (8.3)	3 (8.3)	6 (5.5)	8 (8.0)	4 (4.6)	2 (3.3)	2 (4.8)	1 (7.7)	0 (0.0)	0 (0.0)	0 (0.0)	0 (0.0)	0 (0.0)	29 (5.3)
HPV68	2 (5.6)	2 (5.6)	3 (2.7)	4 (4.0)	4 (4.6)	2 (3.3)	3 (7.1)	0 (0.0)	0 (0.0)	0 (0.0)	0 (0.0)	0 (0.0)	0 (0.0)	20 (3.6)
Multiple HPV types	16 (44.4)	32 (40.5)	32 (29.1)	26 (26.0)	9 (10.3)	10 (16.7)	6 (14.3)	0 (0.0)	3 (30.0)	1 (12.5)	0 (0.0)	0 (0.0)	0 (0.0)	135 (24.6)
HR-HPV gr. A	1 (2.8)	0 (0.0)	0 (0.0)	0 (0.0)	3 (3.4)	2 (3.3)	3 (7.1)	0 (0.0)	0 (0.0)	1 (12.5)	0 (0.0)	0 (0.0)	0 (0.0)	10 (1.8)
HR-HPV gr. B	1 (2.8)	6 (7.6)	8 (7.3)	5 (5.0)	3 (3.4)	5 (8.3)	1 (2.4)	1 (7.7)	1 (10.0)	0 (0.0)	0 (0.0)	0 (0.0)	0 (0.0)	31 (5.7)
Total *n* (%)	36 (100.0)	79 (100.0)	110 (100.0)	100 (100.0)	87 (100.0)	60 (100.0)	42 (100.0)	13 (100.0)	10 (100.0)	8 (100.0)	2 (100.0)	1 (100.0)	0 (0.0)	548 (100.0)

Values are presented as *n* (%). Percentages represent the proportion of positive samples within each age group. HR-HPV = high-risk human papillomavirus. Multiple HPV types indicate co-infection with two or more high-risk HPV genotypes.

**Table 2:** Bethesda system report of HR-HPV genotypes according to liquid-based cytology results.

HR-HPV Result/Type	NILM *n* (%)	ASC-US *n* (%)	LSIL *n* (%)	HSIL *n* (%)	Without LBC Results *n* (%)	Total *n* (%)
HR-HPV (-)	621 (35.5)	105 (6.0)	53 (3.0)	21 (1.2)	399 (22.8)	1199 (68.6)
HPV16	61 (3.5)	12 (0.7)	4 (0.2)	3 (0.2)	40 (2.3)	120 (6.9)
HPV18	13 (0.7)	1 (0.06)	1 (0.06)	1 (0.06)	11 (0.6)	27 (1.5)
HPV31	13 (0.7)	3 (0.2)	3 (0.2)	0 (0.0)	11 (0.6)	30 (1.7)
HPV33	6 (0.3)	0 (0.0)	1 (0.06)	0 (0.0)	12 (0.7)	19 (1.1)
HPV35	1 (0.06)	2 (0.1)	1 (0.06)	1 (0.06)	4 (0.2)	9 (0.5)
HPV39	2 (0.1)	0 (0.0)	0 (0.0)	0 (0.0)	4 (0.2)	6 (0.3)
HPV45	10 (0.6)	5 (0.3)	2 (0.1)	1 (0.06)	6 (0.3)	24 (1.4)
HPV51	7 (0.4)	3 (0.2)	1 (0.06)	1 (0.06)	17 (1.0)	29 (1.7)
HPV52	3 (0.2)	3 (0.2)	1 (0.06)	0 (0.0)	11 (0.6)	18 (1.0)
HPV56	6 (0.3)	3 (0.2)	0 (0.0)	2 (0.1)	11 (0.6)	22 (1.3)
HPV58	0 (0.0)	2 (0.1)	1 (0.06)	0 (0.0)	0 (0.0)	3 (0.2)
HPV59	4 (0.2)	0 (0.0)	1 (0.06)	1 (0.06)	10 (0.6)	16 (0.9)
HPV66	10 (0.6)	5 (0.3)	1 (0.06)	1 (0.06)	12 (0.7)	29 (1.7)
HPV68	7 (0.4)	4 (0.2)	0 (0.0)	1 (0.06)	8 (0.5)	20 (1.1)
>1 HR-HPV	49 (2.8)	19 (1.1)	9 (0.5)	1 (0.06)	57 (3.3)	135 (7.7)
HR-HPV gr. A	10 (0.6)	0 (0.0)	0 (0.0)	0 (0.0)	0 (0.0)	10 (0.6)
HR-HPV gr. B	31 (1.8)	0 (0.0)	0 (0.0)	0 (0.0)	0 (0.0)	31 (1.8)
Total	854 (48.9)	167 (9.6)	79 (4.5)	34 (1.9)	613 (35.1)	1747 (100.0)

Values are presented as *n* (%). Percentages are calculated relative to the total study population (N = 1747), as the denominator, including the 613 (35.1%) patients missing LBC results. HR-HPV = high-risk human papillomavirus; NILM = negative for intraepithelial lesion or malignancy; ASC-US = atypical squamous cells of undetermined significance; LSIL = low-grade squamous intraepithelial lesion; HSIL = high-grade squamous intraepithelial lesion; LBC = liquid-based cytology.

## Data Availability

The data presented in this study are available on request from the corresponding author due to due to reasons of sensitivity. Data are located in con-trolled-access data storage at Medical Diagnostic Laboratory “Synevo”, Bulgaria.
